# Variable Expression of Programmed Cell Death Protein 1-Ligand 1 in Kidneys Independent of Immune Checkpoint Inhibition

**DOI:** 10.3389/fimmu.2020.624547

**Published:** 2021-01-21

**Authors:** Samy Hakroush, Sarah Birgit Kopp, Désirée Tampe, Ann-Kathrin Gersmann, Peter Korsten, Michael Zeisberg, Björn Tampe

**Affiliations:** ^1^Institute of Pathology, University Medical Center Göttingen, Göttingen, Germany; ^2^Department of Nephrology and Rheumatology, University Medical Center Göttingen, Göttingen, Germany; ^3^Department of Nephrology and Rheumatology, German Center for Cardiovascular Research (DZHK), Göttingen, Germany

**Keywords:** programmed cell death protein 1-ligand 1 (PD-L1), checkpoint inhibition, immune-related adverse events (irAE), acute kidney injury, inflammation

## Abstract

**Context:**

Due to recent advantages in cancer therapy, immune checkpoint inhibitors (ICIs) are new classes of drugs targeting programmed cell death protein 1 (PD-1) or its ligand programmed cell death protein 1-ligand 1 (PD-L1) used in many cancer therapies. Acute interstitial nephritis (AIN) is a potential and deleterious immune-related adverse events (irAE) in the kidney observed in patients receiving ICIs and the most common biopsy-proven diagnosis in patients who develop acute kidney injury (AKI). Based on previous reports, AIN in patients receiving ICIs is associated with tubular positivity for PD-L1, implicating that PD-L1 positivity reflects susceptibility to develop renal complications with these agents. It remains unclear if PD-L1 positivity is acquired specifically during ICI therapy or expressed independently in the kidney.

**Methods:**

PD-L1 was analyzed in experimental mouse models of ischemia-reperfusion injury (IRI), folic acid-induced nephropathy (FAN), unilateral ureteral obstruction (UUO), and nephrotoxic serum nephritis (NTN) by immunostaining, SDS-PAGE, and subsequent immunoblotting. In addition, we included a total number of 87 human kidney samples (six renal biopsies with AIN related to ICI therapy, 13 nephrectomy control kidneys, and 68 ICI-naïve renal biopsies with various underlying kidney diseases to describe PD-L1 expression.

**Results:**

We here report distinct PD-L1 expression in renal compartments in multiple murine models of kidney injury and human cases with various underlying kidney diseases, including ICI-related AIN and renal pathologies independent of ICI therapy. PD-L1 is frequently expressed in various renal pathologies independent of ICI therapy and could potentially be a pre-requisit for susceptibility to develop AKI and deleterious immune-related AIN. In addition, we provide evidence that tubular PD-L1 positivity in the kidney is associated with detection of urinary PD-L1^+^ tubular epithelial cells.

**Conclusion:**

Our study implicates that PD-L1 is frequently expressed in various renal pathologies independent of ICI therapy and could potentially be a pre-requisit for susceptibility to develop AKI and deleterious immune-related AIN. Because non-invasive detection of PD-L1^+^ cells in corresponding urine samples correlates with intrarenal PD-L1 positivity, it is attractive to speculate that further non-invasive detection of PD-L1^+^ cells may identify patients at risk for ICI-related AIN.

## Introduction

Due to recent advantages in cancer therapy, immune checkpoint inhibitors (ICIs) are new classes of drugs targeting programmed cell death protein 1 (PD-1) or its ligand programmed cell death protein 1-ligand 1 (PD-L1, synonym CD274, B7 homolog 1) and are used in many cancer therapies (1). By blocking negative co-stimulatory pathways, ICIs allow T cells to remain activated thereby enhancing the anti-tumoral immune response. ICIs are now approved for the use in melanoma, small cell and non-small cell lung cancer, renal cell carcinoma and urothelial carcinoma, among others (1). However, despite benefits with respect to progression-free and overall survival, upregulation of the immune system has been associated with a wide spectrum of systemic immune-related adverse events (irAE) (2). Acute interstitial nephritis (AIN) is a potential and deleterious irAE in the kidney observed in patients receiving ICIs and is the most common biopsy-proven diagnosis in patients developing acute kidney injury (AKI) (3). AIN in patients receiving ICIs is associated with tubular positivity for PD-L1, suggesting that PD-L1 positivity reflects susceptibility to develop renal complications with these agents (3, 4). Additionally to AIN, there is increasing recognition that glomerular disease and renal vasculitis are associated with ICI-related kidney injury with reports describing both, nephrotic and nephritic clinical presentations (5, 6). These observations reveal distinct roles of PD-L1 among different renal compartments dependending on its presence or absence. In the context of ICI-related AKI, PD-L1 expression in distinct renal compartments has not yet been analyzed in human kidneys thus far. Furthermore, it remains still elusive if PD-L1 positivity is acquired specifically during ICI therapy or expressed independently in the kidney. Therefore, we here aimed to compare compartment-specific expression of PD-L1 among ICI-related AIN and ICI-naïve renal pathologies.

## Methods

### Animals

All experimental animal studies were performed with the approval of the Institutional Animal Care and Use Committee of the Beth Israel Deaconess Medical Center and the University Medical Center Göttingen. Experimental protocols are detailed below.

### Ischemia–Reperfusion Injury

Eight to 12 weeks old *C57BL/6* mice were anesthetized with isoflurane inhalation, and analgesia was performed by subcutaneous buprenorphine injection. The left renal hilus was separated from the surrounding tissues and ischemia was performed for 35 min at 25°C ambient room temperature. Mice were sacrificed at indicated time points after ischemic insult as described previously (7).

### Folic Acid-Induced Nephropathy

Kidney injury was induced with a single intraperitoneal injection of folic acid (250 mg/kg body weight in PBS) in *CD1* mice. Mice were sacrificed 96 days after iinjection.

### Unilateral Ureteral Obstruction

Eight to twelve weeks old *CD57BL/6* mice were anesthetized with isoflurane inhalation, analgesia was performed by subcutaneous buprenorphine injection. The ureter was separated from the surrounding tissues and two ligatures were placed about 5 mm apart in the upper two-thirds of the ureter of the left kidney to obtain reliable obstruction. Mice were sacrificed at indicated time points after ureter ligation as described previously (7).

### Nephrotoxic Serum-Nephritis

Each mouse was initially pre-immunized with 200 μg sheep IgG (Capralogics, Gilbertville, USA) in 200 μl complete Freund’s adjuvant (Sigma, St. Louis, USA) and intravenously injected with 40 μl nephrotoxic serum at days 5, 6, and 7 after pre-immunization. Mice were sacrificed 21 days following immunization.

### Human Kidney Specimens

A total number of 87 kidney samples (six renal biopsies with AIN related to ICI therapy, 13 nephrectomy control kidneys and 68 ICI-naïve renal biopsies with various underlying kidney diseases) at the University Medical Center Göttingen were included. The use of parts of kidney biopsies for research purposes was approved by the Ethics Committee of the University Medical Center Göttingen (22/2/14). All patients consented to the use of routinely collected samples and data as part of their regular medical care.

### Immunofluorescence

For immunofluorescent stainings, primary antibodies against PD-L1 (ab205921, Abcam, Cambridge, UK) and Alexa Fluor 488 (Invitrogen, Carlsbad, CA) secondary antibodies were used, nuclear staining was performed using 4′,6-diamidino-2-phenylindole (Vector Laboratories).

### Immunohistochemistry

Formalin-fixed, paraffin-embedded kidney sections were deparaffinized in xylene and rehydrated in ethanol containing distilled water. Tissue sections were stained using antibodies against PD-L1 (ab205921, Abcam, Cambridge, UK), labeling was performed using NovolinkTM Polymer Detection System (Leica Biosystems, Wetzlar, Germany) according to the manufacturer’s protocol. Nuclear counterstain was performed by using Mayer’s Hematoxylin Solution (Sigma, St. Louis, USA).

### Western Blot Analyses

Total kidney lysates were homogenized in NP40 lysis buffer (Invitrogen, Carlsbad, USA) supplemented with protease cocktail inhibitor (Roche, Basel, Switzerland). After sonication, proteins were resolved by Tris-acetate-SDS acrylamide gel electrophoresis and transferred onto PVDF membranes (Invitrogen, Carlsbad, USA) and blocked in 5% dry milk in TBS-T (TBS pH 7.6, 0.1% Tween-20). After incubation with respective primary antibodies against PD-L1 (ab238697, Abcam, Cambridge, UK) and β-actin (ab8227, Abcam, Cambridge, UK) were used following incubation with secondary HRP-conjugated antibodies (Dako, Glostrup, Denmark). Luminescence was detected on an X-ray film using chemiluminescent substrate (Cell Signaling, Danvers, USA). Individual lanes represent biological replicates and densitometry was performed using ImageJ software (National Institute of Health, Bethesda, USA), PD-L1 band density was quantified relative to β-actin.

### Urinary Cytospin

Fresh urine samples were collected and loaded into an automated cytospin machine (Shandon cytospin, Thermo Scientific, Pittsburgh, USA) following the manufacturer’s instructions and centrifuged at 1,000 revolutions per minute (rpm) for 10 min. Slides prepared by cytospin technique were prepared by alcohol-based liquid fixation and stained for Giemsa, CD10 (IR648, Dako, Glostrup, Denmark), EpCAM, WT1 (IR055, Dako, Glostrup, Denmark) and PD-L1 (ab255921, Abcam, Cambridge, UK).

### Flow Cytometry

Antibody staining followed standard protocols for antibody staining of cells in suspension. Within 1 h after collection, urine samples were centrifuged at 1,000 rpm at 4 °C for 8 min. For analysis of urinary cells and KARPAS 299, cell suspensions were washed and resuspended in PBS and stained using antibodies against PD-L1 (329708, Biolegend, San Diego, USA). Cells were incubated with antibody cocktails for 15 min at 4°C in the dark and gated according to positive and isotype negative controls.

### Statistical Methods

Variables were tested for normal distribution using Shapiro-Wilk test. Non-normally distributed continuous variables are expressed as median and interquartile range (IQR), categorical variables are presented as frequency and percentage. Statistical comparisons were not formally powered or prespecified. For group comparisons, the Mann-Whitney U-test was used to determine differences between median values. Spearman correlations were visualized by heatmaps reflecting mean values of Spearman’s Δ, asterisks indicate *p<0.05*. Data analyses were performed with GraphPad Prism (version 8.4.0 for MacOS, GraphPad Software, San Diego, California, USA).

## Results

### Experimental Kidney Injury Induces Programmed Cell Death Protein 1-Ligand 1 Expression in Different Renal Compartments

**Figure 1:**
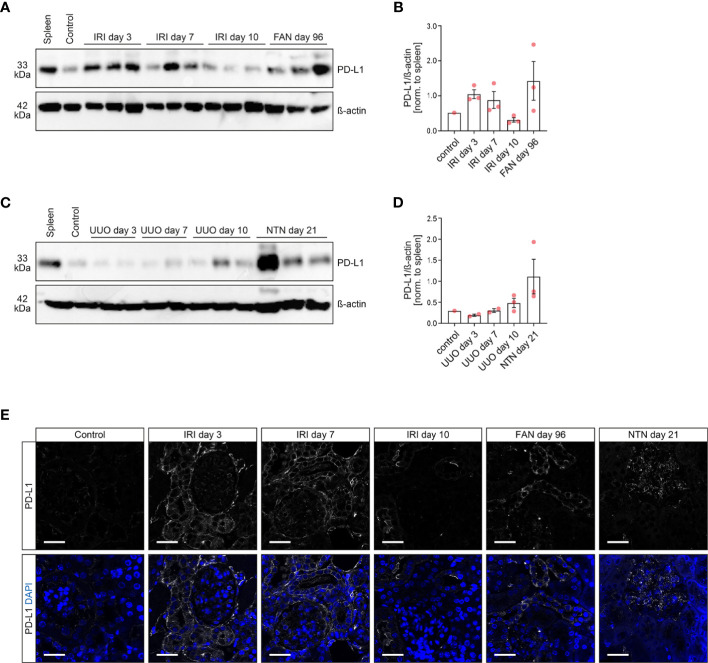
PD-L1 is induced and localizes to different compartments in experimental models of kidney injury. **(A–D)** PD-L1 protein levels were analyzed in multiple models of renal injury by SDS-PAGE and subsequent immunoblotting, individual lanes represent biological replicates. Densitometric analysis of PD-L1/β-actin ratios were normalized to spleen as positive control. **(E)** The panels show representative photomicrographs of kidney sections immunolabeled with primary antibodies against PD-L1 (original magnification x630, scale bar 25 μm). FAN, folic acid-induced nephropathy; IRI, ischemia-reperfusion injury; NTN, nephrotoxic serum nephritis; PD-L1, programmed cell death protein 1-ligand 1; UUO, unilateral ureteral obstruction.

We first analyzed PD-L1 expression in various experimental mouse models of acute kidney injury. By using ischemia-reperfusion injury (IRI) as a model of experimental AKI, we found PD-L1 induced in early injury at day 3 and 7, whereas renal recovery at day 10 after ischemia resulted in normalization of PD-L1 expression (**Figures 1A, B** and **Supplementary Figure 1A**). These observations were further confirmed in folic acid-induced nephropathy (FAN) as a model of AKI primarily due to tubular injury (**Figures 1A, B** and **Supplementary Figure 1A**). By contrast, unilateral ureteral obstruction (UUO) as a model of postrenal failure did not show any PD-L1 regulation wthin the kidney (**Figures 1C, D** and **Supplementary Figure 1B**). In addition, nephrotoxic serum nephritis (NTN) as an established model for experimental glomerulonephritis was equally associated with PD-L1 upregulation (**Figures 1C, D** and **Supplementary Figure 1B**), revealing that damage to distinct compartments of the kidney results in PD-L1 induction. Among different renal compartments, tubular PD-L1 is present in experimental models of acute tubular damage (IRI and FAN), whereas glomerular positivity for PD-L1 was most prominent in a model of glomerular injury (NTN, **Figure 1E**). In summary, we provide evidence that PD-L1 is induced in injured kidneys independently of ICI therapy. Moreover, renal PD-L1 induction depends on the type of kidney injury leading to compartment-specific PD-L1 upregulation.

### Programmed Cell Death Protein 1-Ligand 1 Localizes to Different Compartments in Immune Checkpoint Inhibitor-Related Acute Kidney Injury and Immune Checkpoint Inhibitor-Naïve Kidneys

**Figure 2:**
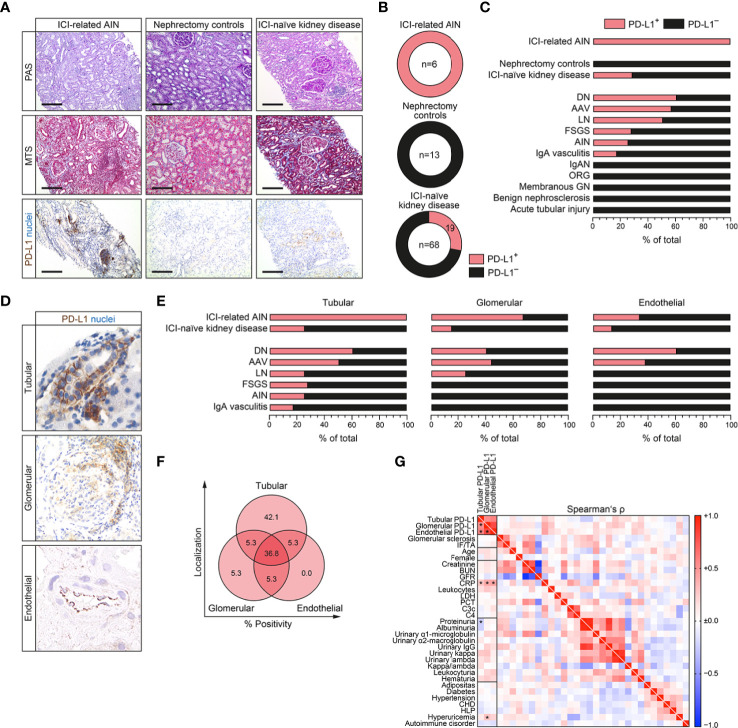
PD-L1 localizes to different compartments in immune checkpoint inhibitor (ICI)-related acute kidney injury (AKI) and ICI-naïve kidneys. **(A–C)** A total number of 87 kidney samples (six renal biopsies with AIN related to ICI therapy, 13 nephrectomy control kidneys and 68 ICI-naïve renal biopsies with various underlying kidney diseases) were analyzed for PD-L1 positivity by immunostaining (original magnification x100, scale bar 200 μm). **(D)** PD-L1 positivity was found in glomerular, tubular and endothelial compartments. **(E, F)** Frequency of PD-L1 positivity among different renal compartments and pathologies. **(G)** Association between PD-L1 status (positive/negative), positivity in tubular, glomerular or endothelial compartments, histopatological, clinical and laboratory findings are shown by heatmap reflecting mean values of Spearman’s Δ, asterisks indicate *p<0.05*. AIN, acute interstitial nephritis; AAV, ANCA-associated vasculitis; BUN, blood urea nitrogen; CHD, coronary heart disease; CRP, C-reactive protein; DN, diabetic nephropathy; FSGS, focal-segmental glomerulosclerosis; GFR, glomerular filtration rate (CKD-EPI); GN, glomerulonephritis; HLP, hyperlipidemia; ICI, immune checkpoint inhibitior; IF/TA, interstitial fibrosis/tubular atrophy; IgAN, IgA nephropathy; LDH, lactate dehydrogenase; LN, lupus nephritis; ORG, obesity-related glomerulopathy; PCT, procalcitonin; PD-L1, programmed cell death protein 1-ligand 1.

Based on our observation that distinct experimental models of tubular and glomerular injury correlate with PD-L1 induction in diseased kidneys, we next included a total number of 87 kidney specimens (6 renal biopsies with AIN related to ICI therapy, 13 nephrectomy control kidneys and 68 ICI-naïve renal biopsies with various underlying kidney diseases, **Supplementary Table 1**). While all nephrectomy control kidneys were negative, PD-L1 expression was found in all cases of ICI-related AKI as reported previously (**Figures 2A–C** and **Supplementary Figure 2A**) (3). Regarding ICI-naïve cases, we could identify 19/68 (27.9%) biopsies from diseased kidneys being positive for PD-L1 (**Figures 2B, C** and **Supplementary Table 2**). PD-L1 positivity was most prominent in diabetic nephropathy (DN), ANCA-associated vasculitis (AAV) and lupus nephritis (LN), but also focal-segmental glomerulosclerosis (FSGS), acute tubulo-interstitial nephritis (AIN) and IgA vasculitis (**Figure 2C**). Among different renal compartments, qualitative histological analyses revealed that tubular PD-L1 was present in all renal pathologies positive for PD-L1, including ICI-related AIN and ICI-naïve cases of kidney disease (**Figures 2D, E**). In contrast, glomerular PD-L1 positivity was only found in ICI-related AIN, DN, AAV and LN (**Figure 2E**). Endothelial PD-L1 expresion was least and only observed in ICI-related AIN, DN and AAV (**Figure 2E**). Comparison of ICI-naïve renal pathologies revealed that only tubular PD-L1 positivity was found in the majority of cases, followed by PD-L1 positivity within all renal compartments (**Figure 2F**). We did not detect isolated endothelial PD-L1, positivity was associated with either glomerular, tubular PD-L1 or both (**Figure 2F**). Correlation analysis with clinical and laboratory findings among ICI-naïve renal pathologies revealed that renal PD-L1 upregulation among all different compartments was consistently associated with elevated C-reactive protein (CRP, **Figure 2G** and **Supplementary Table 3**). Since elevated levels of CRP have been described previously as an early marker to predict irAE in context of ICI therapy, we next analyzed the CRP course among aforementioned cases of ICI-related AIN (8). Baseline and CRP course until AKI development was available in four cases of ICI-related AIN showing no correlation with onset of AKI related to ICI therapy (**Supplementary Figure 2B**), limiting its usability as kidney-specific marker for ICI-related AIN. In summary, we found that PD-L1 positivity is present in all cases of ICI-related AIN and 27.9% of ICI-naïve cases, localized to different renal compartments. Interestingly, elevated levels of CRP were associated with intrarenal PD-L1 positivity in ICI-naïve renal pathologies, whereas no correlation was observed with AKI related to ICI therapy.

### Kidney-Derived Cells Are Detectable in Corresponding Urine Samples Along With Urinary Programmed Cell Death Protein 1-Ligand 1^+^ Cells

**Figure 3:**
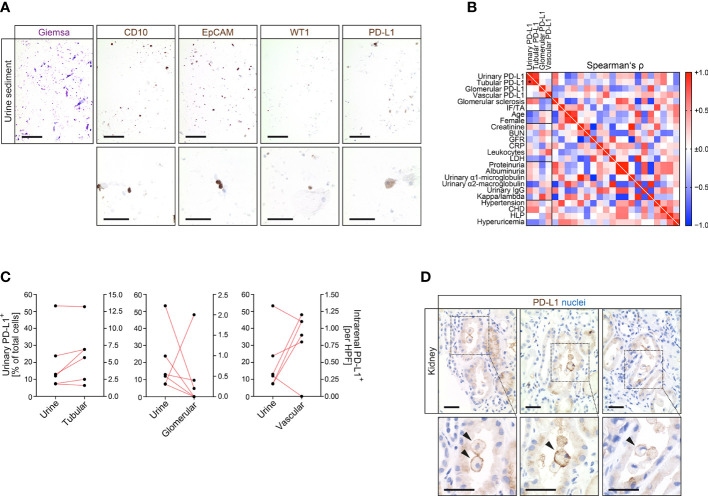
Kidney-derived cells are detectable in corresponding urine samples along with urinary PD-L1^+^ cells. **(A)** Detection of kidney-derived cells including proximal (CD10), distal tubular epithelial cells (EpCAM) and podocytes (WT1) along with PD-L1^+^ cells within urine sediment (upper panel: original magnification x100, scale bar 200 μm; lower panel: original magnification x400, scale bar 20 μm). **(B)** Association between urinary PD-L1^+^ cells and intrarenal PD-L1 positivity in tubular, glomerular or endothelial compartments, histopatological, clinical and laboratory findings are shown by heatmap reflecting mean values of Spearman’s Δ, asterisks indicate *p<0.05*. **(C)** Correlation between urinary PD-L1^+^ cells and PD-L1 positivity in tubular, glomerular and endothelial compartments within corresponding kidneys. **(D)** Detection of PD-L1^+^ cells within renal tubular system in kidney specimens (original magnification x400 with insets are shown, scale bar 25 μm). BUN, blood urea nitrogen; CD10, cluster of differentiation 10; CHD, coronary heart disease; CRP, C-reactive protein; EpCAM, epithelial cell adhesion molecule; GFR, glomerular filtration rate; HLP, hyperlipidemia; IF/TA, interstitial fibrosis/tubular atrophy; LDH, lactate dehydrogenase; PD-L1, programmed cell death protein 1-ligand 1; WT1, Wilms’ tumor 1.

Since intrarenal PD-L1 positivity could potentially be a pre-requisit for susceptibility to deleterious immune-related AIN, we next aimed to directly detect urinary PD-L1^+^ cells in association with intrarenal PD-L1 positivity. Corresponding urine samples of kidney specimens positive for PD-L1 confirmed presence of kidney-derived cells including proximal (CD10^+^), distal tubular epithelial cells (EpCAM^+^) and podocytes (WT1^+^) in addition to PD-L1^+^ cells within urinary sediment (**Figure 3A**). To further validate these observations, we next established flow cytometry for PD-L1^+^ urinary cells. By using KARPAS 299 as established T cell lymphoma cell line positive for PD-L1, PD-L1^+^ cells were equally detectable in corresponding urine samples (**Supplementary Figure 3A, B**) (9). Direct comparison of PD-L1 abundance in the kidney and corresponding urine samples revealed that detection of urinary PD-L1^+^ cells significantly correlated with tubular, but not glomerular or endothelial PD-L1 positivity in the kidney (**Figures 3B, C**). This was further supported by histological observation of detached intratubular PD-L1^+^ epithelial cells in kidney specimens (**Figure 3D**). In summary, we provide evidence that tubular PD-L1 positivity in the kidney correlates with detection of urinary PD-L1^+^ tubular epithelial cells, potentially allowing non-invasive measurement.

## Discussion

This is the first report of distinct PD-L1 expression in renal compartments of multiple murine models and human cases with various underlying kidney diseases, including ICI-related AIN and renal pathologies independent of ICI therapy. AIN is a deleterious immune-related adverse effect in the kidney observed in patients receiving ICIs and the most common biopsy-proven diagnosis in patients who develop AKI (3). On a mechanistic level, AIN in patients receiving ICIs is associated with tubular upregulation of PD-L1, suggesting that PD-L1 positivity reflects susceptibility to develop irAE (3). In addition to AIN, glomerular disease and renal vasculitis have also been described in the context of ICI-related kidney injury (5, 6). These observations implicate distinct roles of PD-L1 among different renal compartments dependent on its presence. We here expand the current knowledge of renal PD-L1 expression within diseased kidneys, to date described only in context of ICI-related AIN. Our observation indicates that PD-L1 is expressed independently of ICI therapy in the kidney. We found PD-L1 positivity in 27.9% of cases with different renal pathologies localized to distinct renal compartments, most prominent in DN, AAV, and LN. This is in line with publicly available transciptome datasets confirming induction of PD-L1 in human CKD samples, microdissected glomeruli in experimental DN and tubulointerstital samples from LN in humans (**Supplementary Figure 4–7**) (10–12). Since PD-L1 positivity has previously been experimentally connected with protection from injury in mice, our findings implicate that PD-L1 is equally induced as a protective, anti-inflammatory response to injury in diseased human kidneys (13, 14). Furthermore, we provide evidence that PD-L1 expression in the kidney is associated with elevated levels of CRP, in line with previous reports that elevated levels of CRP are an early marker to predict irAE in the context of ICI therapy (8). While levels of CRP did not correlate with AKI related to ICI therapy, renal PD-L1 positivity in the kidney correlates with detection of PD-L1^+^ urinary cells, thereby allowing non-invasive measurement. In addition, ICI therapy may be the only therapeutic option available to effective treat and maintain tumor remission in certain cancers. A key concern is the safety of ICI re-challenge after an episode of AKI. Based on previous reports, recurrence of ICI-related AKI occurred in about 23% of rechallenged patients, with a shorter latency period between the initial AKI episode and rechallenge (15). Therefore, it is also tempting to speculate that measurement of shedded PD-L1^+^ renal cells may also allow non-invasive monitoring of rechallenged patients to detect recurrence of ICI-related AKI. These assumptions have to be corroborated further.

In summary, our study implicates that PD-L1 is frequently expressed in various renal pathologies independent of ICI therapy and could potentially be a pre-requisit for susceptibility to develop AKI and deleterious immune-related AIN. Since non-invasive detection of PD-L1^+^ cells in corresponding urine samples correlates with intrarenal PD-L1 positivity, it is attractive to speculate that further non-invasive detection of PD-L1^+^ cells may identify patients at risk for ICI-related AIN.

## Data Availability Statement

The original contributions presented in the study are included in the article/**Supplementary Material**, further inquiries can be directed to the corresponding author.

## Ethics Statement

The studies involving human participants were reviewed and approved by the Ethics Committee of the University Medical Center Göttingen. The patients/participants provided their written informed consent to participate in this study. The animal study was reviewed and approved by the Institutional Animal Care and Use Committee of the Beth Israel Deaconess Medical Center and the University Medical Center Göttingen.

## Author Contributions

BT conceived the study, collected and analyzed the data, and co-wrote the first draft. SH, SK, DT, and AG collected and analyzed the data. PK and MZ participated in the construction and editing of the manuscript. SH and SBK contributed equally as first co-authors. All authors contributed to the article and approved the submitted version.

## Funding

BT was supported by the Research program, University Medical Center Göttingen (1403720 to BT). This work was also supported by grants from the Deutsche Forschungsgemeinschaft (ZE523/2-3 and SFB1002/D03 to MZ) and by equipment grant INST 1525/16-1. We also acknowledge support by the Open Access Publication Funds of the Göttingen University. The funding sources had no involvement in the design, collection, analysis, interpretation, writing, or decision to submit the article.

## Conflict of Interest

The authors declare that the research was conducted in the absence of any commercial or financial relationships that could be construed as a potential conflict of interest.
