# Supplementary material for: Variable Expression of Programmed Cell Death Protein 1-Ligand 1 in Kidneys Independent of Immune Checkpoint Inhibition
Source: Front Immunol. 2021 Jan 21;11:624547. doi: 10.3389/fimmu.2020.624547 (PMC7858644; doi:10.3389/fimmu.2020.624547)
Supplement: Supplementary file 1 [file DataSheet_1.pdf]

Variable Expression of Programmed Cell Death Protein 1-Ligand 1 in Kidneys Independent of Immune Checkpoint Inhibition

Supplementary Material

Samy Hakroush[1], Sarah Birgit Kopp[2], Désirée Tampe[2], Ann-Kathrin Gersmann[1], Peter Korsten[2], Michael Zeisberg[2,3], Björn Tampe[2]

[1]*Institute of Pathology, Göttingen University Medical Center, Georg August University, Göttingen, Germany*

[2]*Department of Nephrology and Rheumatology, University Medical Center Göttingen, Georg August University, Göttingen, Germany*

[3]*Department of Nephrology and Rheumatology, German Center for Cardiovascular Research (DZHK), Göttingen, Germany*

Corresponding author:

Björn Tampe, MD

Department of Nephrology and Rheumatology

University Medical Center Göttingen

Georg August University

Göttingen, Germany

Email: bjoern.tampe@med.uni-goettingen.de

Supplementary Table 1. Characteristics of patients with ICI-related AKI.

	Case #1	*Case #2*	*Case #3*
ICI therapy	Nivolumab	Nivolumab	Durvalumab
Type of cancer disease	Melanoma	Melanoma	NSCLC
AKI stage	3	3	2
AKI – days after ICI	97	140	182
Maximum creatinine – µmol/L	579.9	578.1	199.8
Urinary findings			
Proteinuria– mg/L	150.0	153.8	115.4
α_1-microglobulin – mg/L	51.9	29.3	7.8
IgG – mg/L	21.5	9.6	ND
uACR – mg/g	214.96	114.47	13.53
Renal histology			
Tubulointerstitial	AIN	AIN	AIN
Glomerular	Podocyte injury	Activated podocyte	Glomerular hypertrophy
Fibrosis – (%)	50	40	30
	Case #4	*Case #5*	*Case #6*
ICI therapy	Pembrolizumab	Nivolumab	Pembrolizumab
Type of cancer disease	NSCLC	Melanoma	Oral cancer
AKI stage	2	2	3
AKI – days after ICI	128		
Maximum creatinine – µmol/L	214.8	370.4	293.5
Urinary findings			
Proteinuria– mg/L	372.6	479.5	1522.4
α_1-microglobulin – mg/L	67	72	82.6
IgG – mg/L	5.3	21.9	131
uACR – mg/g	44.46	204.92	673.32
Renal histology			
Tubulointerstitial	AIN	AIN	AIN
Glomerular	Podocyte effacement	Podocyte injury	Glomerular nephritis
Fibrosis – (%)	40	10	5

Abbreviations: AIN, acute interstitial nephritis; AKI, acute kidney injury; ICI, immune checkpoint inhibitor; ND, not determined; NSCLC, non small-cell lung cancer; uACR, urinary albumin/creatinine ratio.

Supplementary Table 2. Characteristics of patients positive versus negative for PD-L1 in ICI-naïve kidneys.

	Positive for PD-L1 (n=19)	*Negative for PD-L1 (n=49)*
Female sex (%) – no.	8 (42.1)	21 (42.9)
Median age (IQR) – years	69 (53-77)	58 (42-70.5)
Renal injury		
Median serum creatinine (IQR) – µmol/L	197 (113-307)	153 (96.8-295)
Median BUN (IQR) – mmol/L	35 (15-58)	34 (19-44)
Median GFR (IQR) – mL/min/1.73m^2	29 (20-60)	33 (20-59.4)
Laboratory findings		
Median CRP (IQR) – mg/L	46.5 (4.6-77.5)	10.5 (2.2-30.1)
Median leukocytes (IQR) – x1000/µL	10.7 (6.8-17)	8.2 (6.4-10.9)
Median LDH (IQR) – U/L	292 (238-350)	293 (249-364)
Median PCT (IQR) – µg/L	0.25 (0.15-0.39)	0.2 (0.1-0.61)
Median C3c (IQR) – g/L	1.2 (0.8-1.4)	1.3 (1.1-1.4)
Median C4 (IQR) – g/L	0.27 (0.11-0.35)	0.28 (0.22-0.36)
Urinary findings		
Median proteinuria (IQR)– mg/L	430 (99.9-1996)	908 (295-1833)
Median albuminuria (IQR) – mg/L	178 (12.4-783)	460 (82.3-1110)
Median α_1-microglobulin (IQR) – mg/L	31.4 (2.5-79.5)	23 (9-71.2)
Median α_2-macroglobulin (IQR) – mg/L	3 (2.7-6.5)	2.7 (2.7-9.8)
Median IgG (IQR) – mg/L	38.3 (12.9-262)	46.2 (14.9-151)
Median kappa – mg/L	91.3 (24.5-138)	46.3 (16.5-86.7)
Median lambda (IQR) – mg/L	38.4 (9.7-76.2)	21.6 (9.3-46.7)
Median kappa/lambda (IQR)	1.9 (1.7-3.5)	1.9 (1.6-2.2)
Leukocyturie (%) – no.	10 (52.6)	31 (63.3)
Hematuria (%) – no.	11 (57.9)	26 (53.1)
Renal histology		
Median glomerular sclerosis – (%)	18.5 (0-27.5)	14.8 (0-48.2)
Median IF/TA – (%)	40 (0-50)	20 (5-50)
Comorbidities		
Adipositas (%) – no.	7 (36.8)	16 (32.7)
Diabetes (%) – no.	5 (26.3)	8 (16.3)
Hypertension (%) – no.	12 (63.2)	30 (61.2)
CHD (%) – no.	2 (10.5)	7 (14.3)
HLP (%) – no.	2 (10.5)	9 (18.4)
Hyperuricemia (%) – no.	7 (36.8)	8 (16.3)
Autoimmune disorder (%) – no.	9 (47.4)	28 (57.1)

Abbreviations: BUN, blood urea nitrogen; CHD, coronary heart disease; CRP, C-reactive protein; GFR, glomerular filtration rate; HLP, hyperlipidemia; ICI, immune checkpoint inhibitir; IF/TA, interstitial fibrosis/tubular atrophy; IQR, interquartile range; LDH, lactate dehydrogenase; No., number; PCT, procalcitonin; PD-L1, programmed cell death protein 1-ligand 1.

Supplementary Table 3. Characteristics of patients with tubular, glomerular or vascular PD-L1 positivity in ICI-naïve kidneys.

	Tubular PD-L1	*Glomerular PD-L1*	*Vascular PD-L1*
Female sex (%) – no.	6 (35.3)	6 (60)	5 (55.6)
Median age (IQR) – years	69 (49-78.5)	71 (51.8-80)	63 (47.3-78)
Renal injury			
Median serum creatinine (IQR) – μmol/L	1897(114-301)	283 (76.9-349)	283 (71.6-402)
Median BUN (IQR) – mmol/L	35 (15-57.5)	42 (14.5-76.3)	32 (13.5-65)
Median GFR (IQR) – mL/min/1.73m^2	29 (20-58.1)	21.1 (20-60)	21.1 (20-60)
Laboratory findings			
Median CRP (IQR) – mg/L	46.5 (4.7-58.8)	57.4 (40.3-149)	57.4 (48.7-133)
Median leukocytes (IQR) – x1000/μL	10.6 (6.8-16)	12.4 (7.9-19.1)	9.9 (7.7-20)
Median LDH (IQR) – U/L	293 (239-357)	279 (237-357)	314 (257-414)
Median PCT (IQR) – μg/L	0.29 (0.12-0.43)	0.25 (0.19-0.36)	0.28 (0.17-0.38)
Median C3c (IQR) – g/L	1.3 (0.84-1.4)	1.3 (0.98-1.4)	1.4 (1.1-1.5)
Median C4 (IQR) – g/L	0.27 (0.15-0.35)	0.27 (0.15-0.35)	0.28 (0.24-0.35)
Urinary findings			
Median proteinuria (IQR)– mg/L	338 (95.6-1555)	984 (342-3784)	984 (428-3432)
Median albuminuria (IQR) – mg/L	173 (8.8-479)	282 (102-1803)	282 (156-1721)
Median α_1-microglobulin (IQR) – mg/L	22.2 (2.2-77.6)	70.3 (21.8-90.2)	70.7 (15.3-102)
Median α_2-macroglobulin (IQR) – mg/L	3.4 (2.7-8.1)	2.9 (2.7-5.7)	3.8 (2.7-7.4)
Median IgG (IQR) – mg/L	29.2 (11.4-220)	102 (29.2-343)	102 (30.2-334)
Median kappa – mg/L	91.3 (24.4-136)	91.3 (43.3-135)	88.3 (24.5-93.5)
Median lambda (IQR) – mg/L	38.4 (6.6-71.3)	38.4 (21.3-71.8)	38.4 (12.9-62.6)
Median kappa/lambda (IQR)	2.1 (1.6-3.9)	1.8 (1.5-3.6)	1.8 (1.5-2.7)
Leukocyturie (%) – no.	9 (52.9)	8 (80)	7 (77.8)
Hematuria (%) – no.	10 (58.8)	8 (80)	6 (75)
Renal histology			
Median glomerular sclerosis – (%)	18.5 (0-37.5)	20.1 (0-62.5)	17.6 (0-84.4)
Median IF/TA – (%)	40 (0-50)	47.5 (0-62.5)	47.5 (1.3-65)
Comorbidities			
Adipositas (%) – no.	6 (35.3)	3 (30)	4 (44.4)
Diabetes (%) – no.	4 (23.5)	2 (20)	1 (11.1)
Hypertension (%) – no.	10 (58.8)	6 (60)	6 (66.7)
CHD (%) – no.	1 (5.9)	1 (10)	1 (11.1)
HLP (%) – no.	1 (5.9)	1 (10)	1 (11.1)
Hyperuricemia (%) – no.	5 (29.4)	5 (50)	4 (44.4)
Autoimmune disorder (%) – no.	7 (41.2)	4 (40)	3 (33.3)

Abbreviations: BUN, blood urea nitrogen; CHD, coronary heart disease; CRP, C-reactive protein; GFR, glomerular filtration rate; HLP, hyperlipidemia; ICI, immune checkpoint inhibitir; IF/TA, interstitial fibrosis/tubular atrophy; IQR, interquartile range; LDH, lactate dehydrogenase; No., number; PCT, procalcitonin; PD-L1, programmed cell death protein 1-ligand 1.

Supplementary Figure 1

A **B**

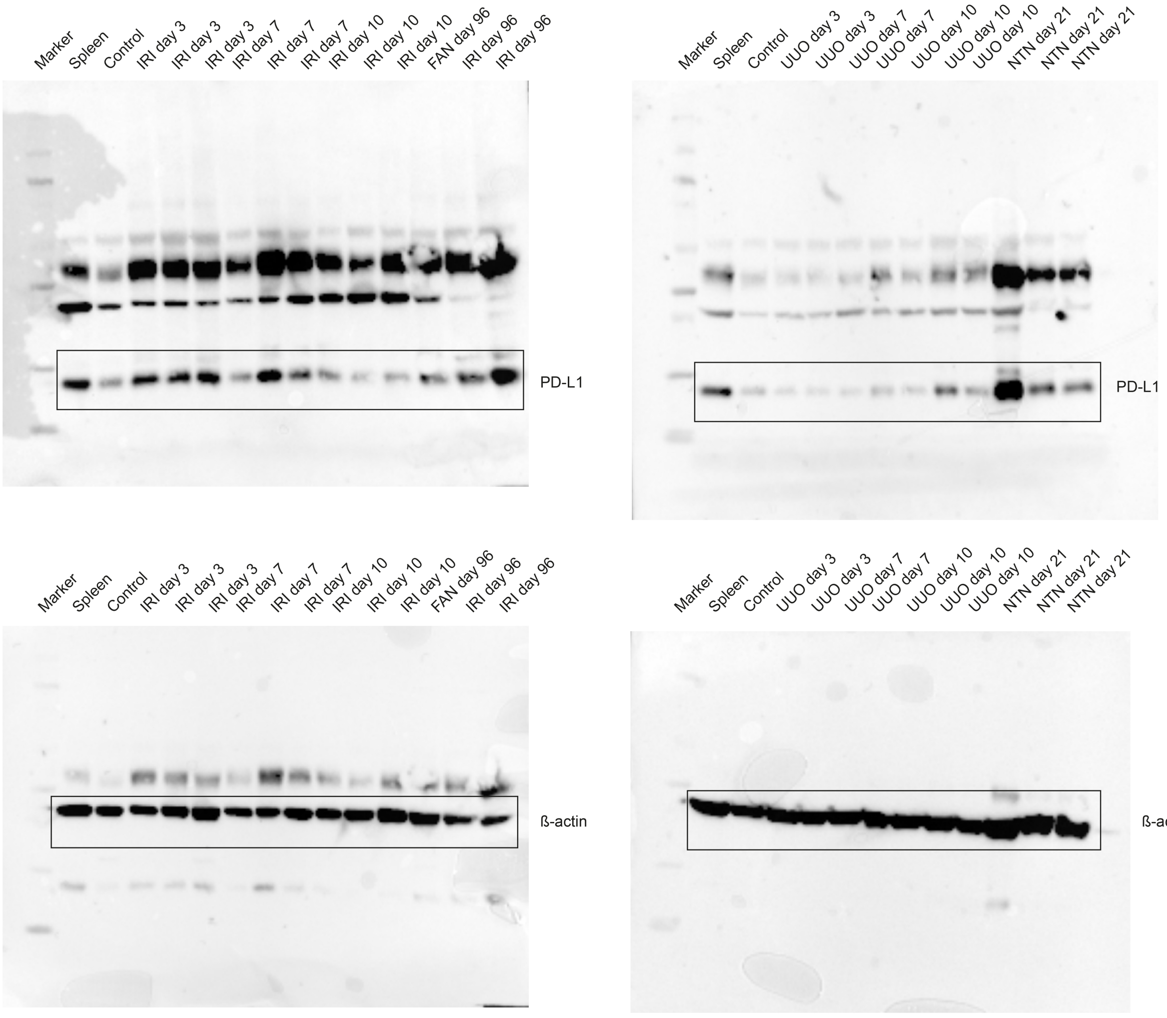

Supplementary Figure 1. (**A,B**) PD-L1 protein levels were analyzed in multiple models of renal injury by SDS-PAGE and subsequent immunoblotting, uncropped gel images corresponding to Figure 1A,B are shown.

Supplementary Figure 2

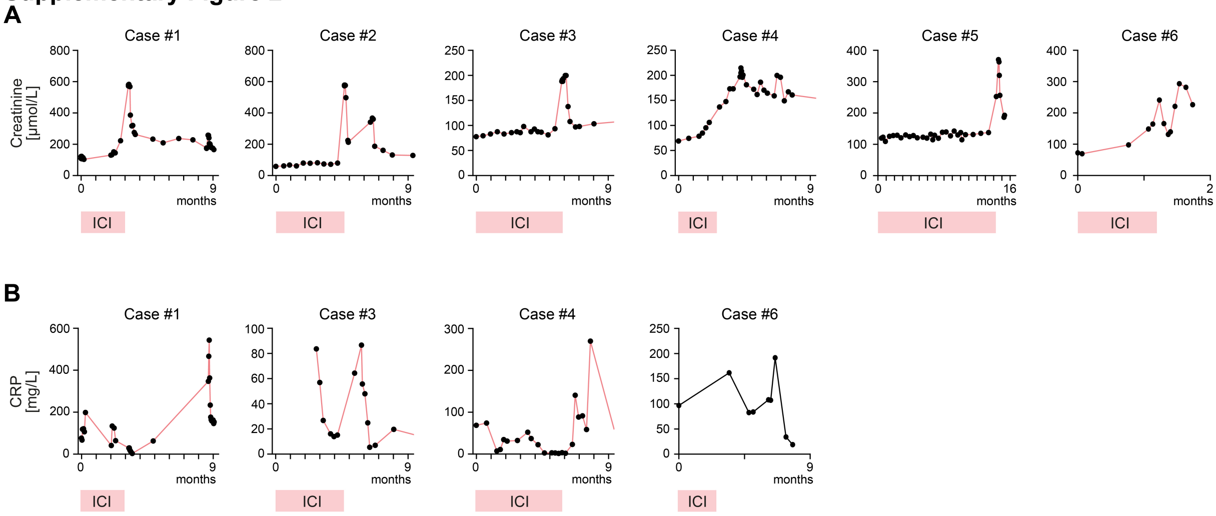

Supplementary Figure 2. (**A**) Plasma creatinine levels and onset of AKI among cases with ICI therapy. (**B**) Course of CRP levels among cases with AKI related to ICI-therapy. Abbreviations: AIN, acute interstitial nephritis; AKI, acute kidney injury; CRP, C-reactive protein; HE, hematoxylin and eosin; ICI, immune checkpoint inhibitor; PAS, periodic acid-Schiff; PD-1, programmed cell death protein 1; PD-L1, programmed cell death protein 1-ligand 1.

Supplementary Figure 3

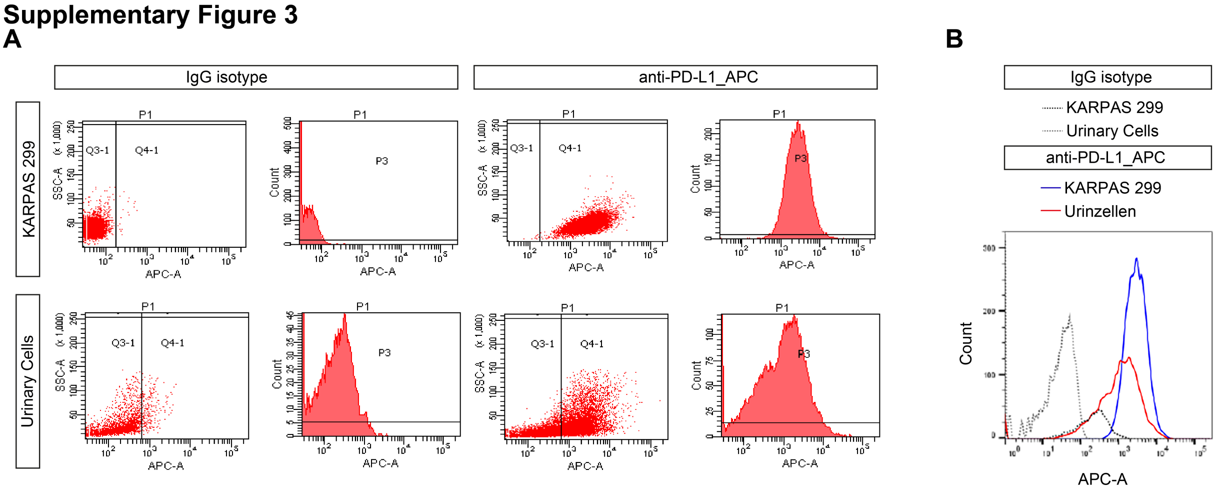

Supplementary Figure 3. (**A,B**) Flow cytometry for PD-L1^{+} urinary cells compared to KARPAS 299 as established T cell lymphoma cell line positive for PD-L1.

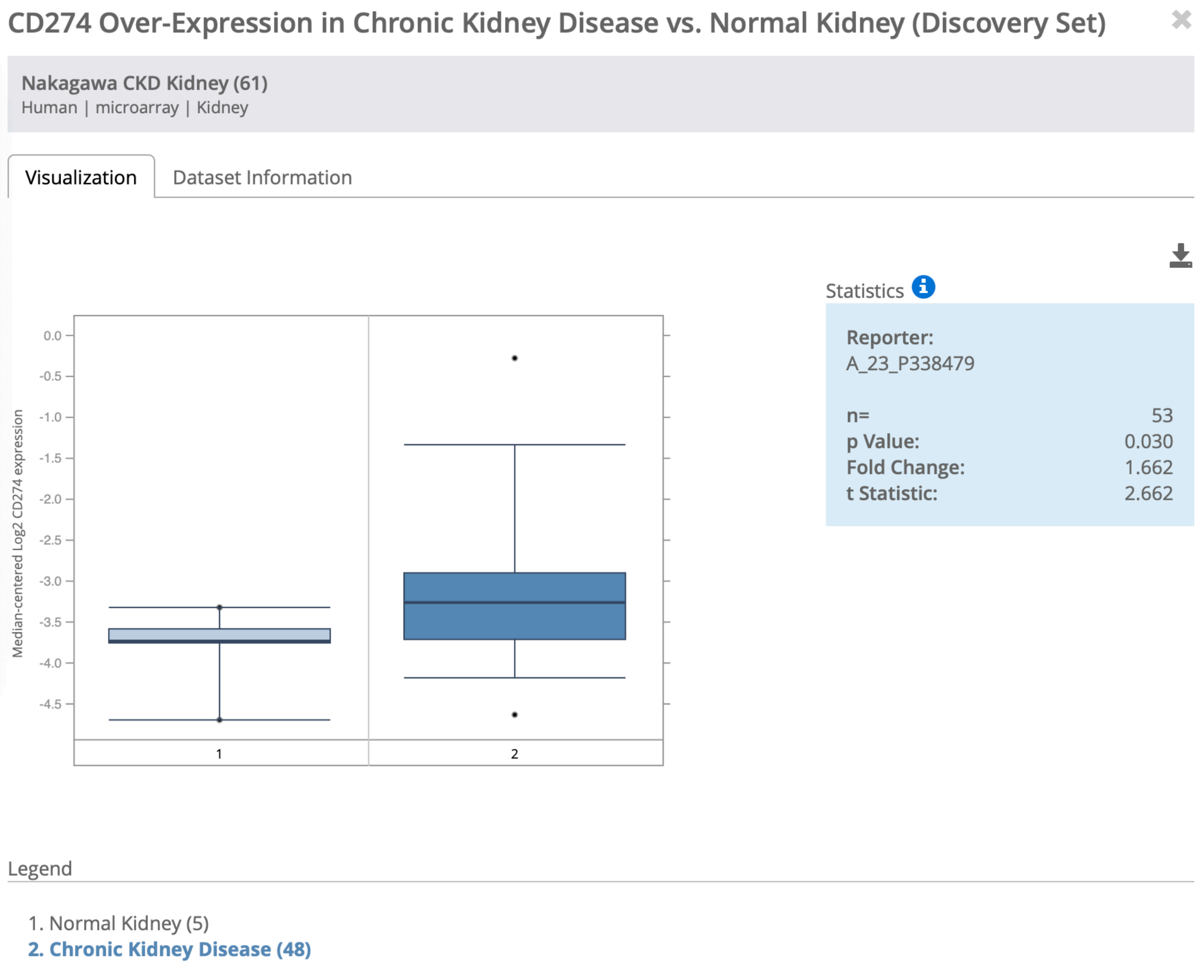

Supplementary Figure 4. Data extracted from NephroSeq (references 10,12).

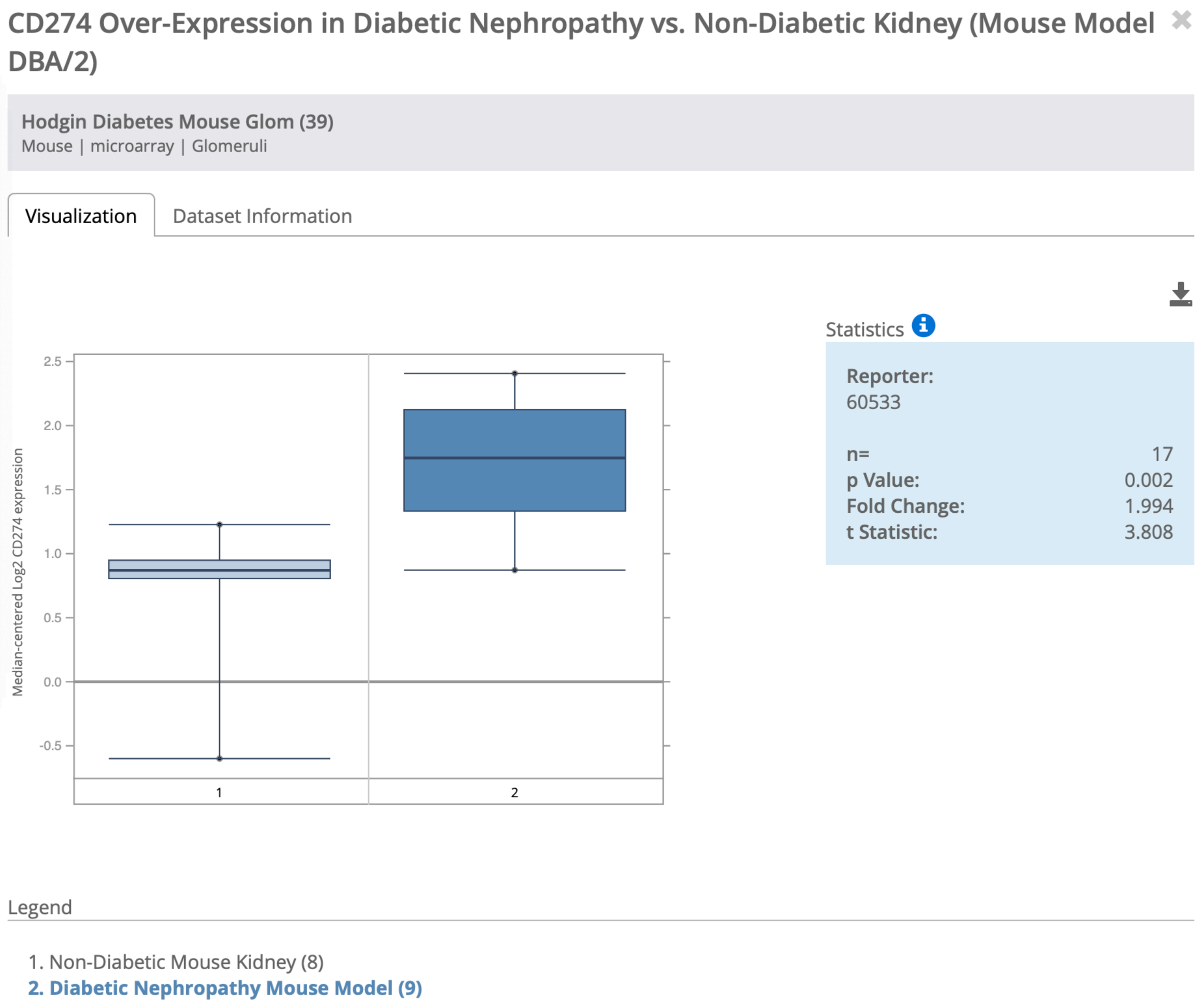

Supplementary Figure 5. Data extracted from NephroSeq (references 11,12).

CD274 Over-Expression in Diabetic Nephropathy vs. Non-Diabetic Kidney (Mouse Model db/db C57BLKS)

Hodgin Diabetes Mouse Glom (39)
Mouse | microarray | Glomeruli

Visualization   Dataset Information

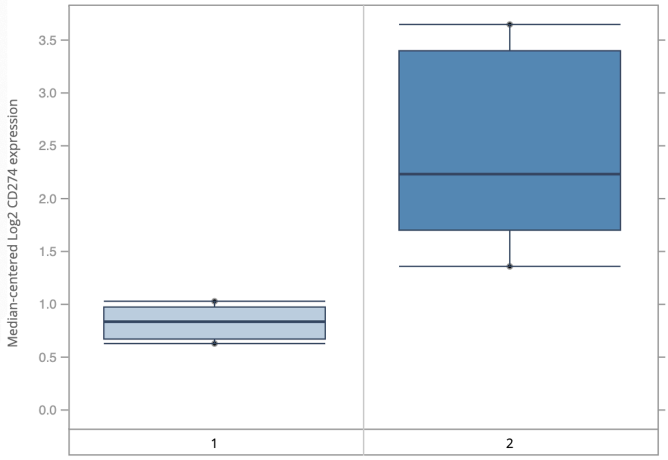

Statistics

Reporter:
60533

**n=**	10
**p Value:**	0.021
**Fold Change:**	3.117
**t Statistic:**	3.554

Legend

1. Non-Diabetic Mouse Kidney (5)
2. **Diabetic Nephropathy Mouse Model (5)**

Supplementary Figure 6. Data extracted from NephroSeq (references 11,12).

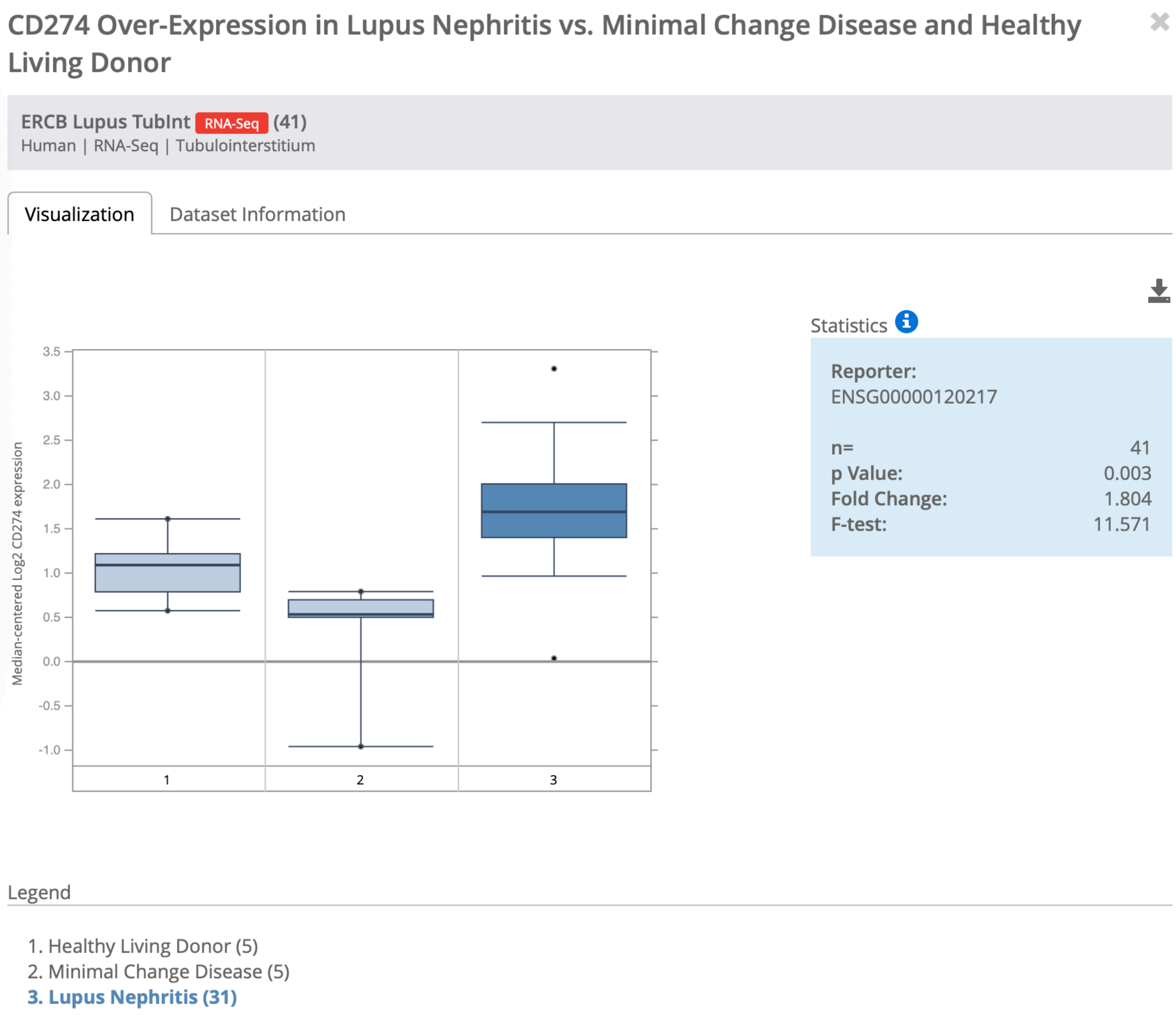

Supplementary Figure 7. Data extracted from NephroSeq (references 12).
